# A Multi-Criteria Framework for Pandemic Response Measures

**DOI:** 10.3389/fpubh.2021.583706

**Published:** 2021-04-20

**Authors:** Love Ekenberg, Adriana Mihai, Tobias Fasth, Nadejda Komendantova, Mats Danielson

**Affiliations:** ^1^International Institute for Applied Systems Analysis, IIASA, Laxenburg, Austria; ^2^Department of Computer and Systems Sciences, Stockholm University, Kista, Sweden; ^3^Innovating Governance, Vienna, Austria; ^4^Centre of Excellence for the Study of Cultural Identity, University of Bucharest, Bucharest, Romania; ^5^Department of Public Health Analysis and Data Management, Public Health Agency of Sweden, Solna, Sweden

**Keywords:** multi-criteria decision analysis, managing and mitigating the risk of COVID 19 pandemic, risk governance, SEIR models, participation and inclusion

## Abstract

In managing the COVID-19 pandemic, several compelling narratives seem to have played a significant role in the decision-making processes regarding which risk mitigation and management measures to implement. Many countries were to a large extent unprepared for such a situation, even though predictions about a significant probability for a pandemic to occur existed, and national governments of several countries often acted in an uncoordinated manner, which resulted in many inconsistencies in the disaster risk reduction processes. Limited evidence has also made room for strategic narratives meant to persuade the public of the chosen set of actions, even though the degree of uncertainty regarding the outcomes of these was high, further complicating the situation. In this article, we assume a normative standpoint regarding rhapsodic decision making and suggest an integrated framework for a more elaborated decision analysis under the ambiguity of how to contain the virus spread from a policy point of view, while considering epidemiologic estimations and socioeconomic factors in a multi-stakeholder-multi-criteria context based on a co-creative work process for eliciting attitudes, perceptions, as well as preferences amongst relevant stakeholder groups. The framework, applied in our paper on Romania for demonstrative purposes, is used for evaluating mitigation measures for catastrophic events such as the COVID-19 situation, to mobilize better response strategies for future scenarios related to pandemics and other hazardous events, as well as to structure the production and analysis of narratives on the current pandemic effects.

## Introduction

The recent emergence of the COVID-19 pandemic situation highlighted that many countries have to a large extent been unprepared for it (1). Decision-makers had to operate in conditions of severe uncertainty about the case fatality rate, the spreading of the virus, the timing of infectiousness, the number of asymptomatic cases—just to mention a few (2). Risk mitigation measures such as vaccines were missing (3) and decision-makers did not have reliable information about critical measures to protect society from the virus spread or at least to reduce its exposure and vulnerability. Another critical problem in assessing the risk was that the evidence about the case fatality rate was unknown (4). As a result of this and many other factors during the COVID-outbreak (5), public authorities had to make decisions based on uncertain quantitative evidence and expert scientific advice (e.g., about possible future scenarios), on assessments of the health system capacity (especially of intensive care units), on expected public adoption of more or less restrictive measures, and on the evolution of national public debates about the issue (6). Nevertheless, the disaster risk reduction of the COVID-19 pandemic showed that in deciding which measures to implement, many countries acted in an apparently uncoordinated manner, at least at the beginning of the pandemic. The measures undertaken by bordering countries or regions within one country were many times inconsistent, and decisions on whether or not to impose lockdown were not taken only based on the number of confirmed cases. The effects of these inconsistencies are to a large extent still unforeseeable. Moreover, many non-pharmaceutical measures are progressively limiting individual freedom and have high economic and societal costs when undertaken with the aim to avoid fatalities in the short term, even though the same measures might produce indirect long-term fatalities due to economic recession and restricted access to healthcare by non-COVID-19 patients, restricted access to education (7) and other effects upon a large number of socioeconomic factors.

Furthermore, limited evidence has made room for strategic narratives meant to persuade the public of a chosen set of actions even though the degree of uncertainty regarding the outcomes of these was high. These narratives have explanatory power, reducing the cognitive overload of information, but also mobilizing power, in particular in crisis communication where stories that give a sense of collective action, such as to fight against a threat, are preferred. This threat is often framed by assigning blame to various actors so as to stir anger (against, for instance, China or the novel coronavirus itself), or by using aggressive representations of the threat and its possible impact to stir fear (8). The latter include early media reports on Lombardy and Wuhan as well as the “flatten the curve” visual metaphor indicating how the estimated numbers of cases exceed the limits of the sanitary systems if more aggressive social distancing measures are not implemented. In crisis communication, some of the downsides of this strategy, including social and political polarization, oversimplification of the problem, and anxiety and other negative psychological reactions, are traded for the benefit of the proposed course of action which needs to be adopted by the public. However, without a clear estimate of the benefits of the chosen set of measures, the use of persuasive narratives can be unjustified and trigger mistrust in the communicators, as well as low compliance rates to the current and future measures to mitigate the pandemic.

Several cognitive and behavioral biases seem also to have played a role in the decision-making processes. One such is connected with risk perceptions under conditions of ambiguity (9). A probable component is also bounded rationality, when individuals are limited regarding their ability or willingness to collect information and are unable to identify an even perceived optimal solution, leading to decisions being made in a significantly simplified decision space. Decision-makers thus search in this sense for a satisfactory solution, but they focus only on a limited set of options from available alternatives (10, 11). Then there is an inevitable component of dread risk (compare, e.g., with hazardous technologies) connected with the people' judgments about unknown risks and their “perceived lack of control, dread, catastrophic potential, fatal consequences, and the inequitable distribution of risks and benefits” (12).

We do not criticize the adopted measures *per se*, but rather the existing decision-making mechanisms under conditions of uncertainty, where reliable data is scarce and the impact of the chosen policy across a variety of interconnected sectors and social categories is potentially quite serious. Rather than looking only at epidemiologic and healthcare factors, our purpose is to expand the policy problem and to include socioeconomic factors as well in the decisions over measures to be adopted in response to the pandemic, since the consequences of any chosen policy upon a variety of fields and groups need to be carefully and transparently weighed.

In this paper, we present a framework for decision analysis under ambiguity on how to treat the virus spread from a policy point of view. Our framework takes into consideration both epidemiologic estimations and socioeconomic factors and could also provide an enabler for strategic communication in the public sphere and facilitate a discussion about a range of policies, even in contexts of strong uncertainty. A main part of this is a multi-stakeholder-multi-criteria framework for eliciting attitudes, perceptions and preferences amongst relevant stakeholder groups. The decision process is based on a recognition of the complex relationships between different criteria and is supposed to support national and local strategies in dealing with pandemic emergencies and action plans, allowing for an alignment of overall objectives with perceptions and preferences of various stakeholder groups on priorities. Since there is a heterogeneity of opinions and potential conflicts of various stakeholders about disaster risk reduction measures, the recommendations should be based on compromise solutions to increase the quality, acceptability and legitimacy of the decision-making processes.

In the following sections, we first provide an overview of the various non-pharmaceutical measures put in place in order to mitigate the pandemic, in which we trace a number of inconsistent emergency responses and the gaps in estimating measures' impacts. Since uncertainty in epidemiologic data and projections has been shown to be a pervasive problem in current risk estimations, we then present a decision analytical framework that can be used under conditions of severe uncertainty, which presupposes the implementation of participatory components and a formalized evaluation process of the possible measures which can be adopted. We then exemplify how our framework can be applied at a national level, using Romania as a case study, for which we use an augmented SEIR model for epidemiologic data estimations, publicly available official reports for socioeconomic data and a stakeholder questionnaire for showcasing priority ranking in pandemic responses.

## Measures under uncertainty

Measures to contain the spread of the novel SARS-CoV-2 virus have to a large extent been based on various epidemiologic risk assessments, which were made primarily by centers of disease control and prevention in Europe and the USA and by the World Health Organization, as well as by various consultants and trusted parties (13). These assessments established scenarios starting from the number of confirmed infections in a country, with every scenario having a series of recommendations on containment measures to use in order to limit the spread of the virus. There are, however, challenges with modeling the effects of risk mitigation measures. Many epidemiologic models do not take into consideration demographics, distribution of population, age groups and their interaction patterns. Furthermore, there is limited evidence included in currently used models (14) on how each measure reduces the rate of transmissibility. The assumptions which serve as a basis for predictions are that there is no change in behavior and that preventive measures are put in place at one specific time-point. Then time calibration is done using the observed number of case fatalities and estimates of the time between infection and death and the infection fatality risk. It is also assumed that the overall effect of preventive measures is known. The effects are estimated from the observed increased doubling time after preventive measures are put in place. However, the predictions are highly sensitive to the doubling times without and with preventive measures, as well as to, for instance, the reproduction number, but less sensitive to the estimates used for time-calibration: observed number of case fatalities, the typical time between infection and death, and the infection fatality risk (15).

Aside from the increased healthcare and treatment optimization efforts, non-pharmaceutical interventions are layered progressively, starting from more low-cost measures (increasing personal hygiene through hand-washing, disinfecting surfaces and wearing face masks), to isolating individuals confirmed positive with the virus, to, eventually, more aggressive and costly social distancing measures. Countries have taken different approaches as to which set of measures to introduce and when, which of course is difficult given the uncertainty regarding the time frame for containing the pandemic, how much the economy can sustain the associated costs of social distancing and isolation measures, as well as the uncertainty of how long citizens can comply with certain measures.

For instance, in Romania, previous risk assessments on severe flu epidemic scenarios made in 2016 (16), considered a novel flu virus strain, with an attack rate of 35% (higher among children), a case fatality rate between 0.4 and 1.2%, leading to 30,000 hospitalizations and more than 1,000 deaths among the vulnerable age groups: people of all ages suffering from chronic illnesses, healthcare system employees, social protection facilities' employees and residents, and elderly people. In terms of impact, the health and healthcare costs were considered very high, while the economic costs were estimated to have a medium impact of 101–500 million Euro (0.03 of GDP). The non-pharmaceutical measures to contain this epidemic scenario included possible school closures affecting, for more than a week, 500,000 students at most, temporary workplace disruptions affecting mostly 500,000 employees and postponements of cultural and sports events. In contrast, between March 2020 and July 2020, the partial lockdown measures taken to contain the novel coronavirus in Romania severely affected 900,000 primary and secondary school students with no access to education for 4 months (17), led to over 900,000 suspended work contracts and almost 300,000 unemployed. The economic costs associated with the measures had an impact of over 4 billion euro (1.7–1.9 of GDP) by July 2020 (18, 19), at a time when in Romania there were 1,900 deaths caused by COVID-19, the majority of confirmed cases (over 40,000) being however asymptomatic or mild. The uncertainty of epidemiologic evidence transferred to general uncertainty about policy impact, leading to much higher socioeconomic costs than the ones previously envisioned in case of a severe epidemic.

Some countries, such as Japan, have mainly focused on contact tracing and testing, recommending people to restrict their travels, and teach and work from home. Sweden chose to cancel larger public events, but did not close primary schools and workplaces, while the idea of keeping social distance has been largely promoted. South Korea had a similar approach, but with more intensive contact tracing using digital systems. Interestingly, Taiwan, in spite of its proximity to China, had one of the lowest stringency levels (20), as they did not close down schools, workplaces or public transport, and instead mostly focused on tracing and isolating measures. Taiwan's experience with the 2003 SARS epidemic could account for a series of quick decisions involving travelers' screening, a wide distribution of masks, hand sanitizers and thermometers (21), as well as the investment of ~$6.8 million into the manufacturing sector to create 60 new mask production lines.

There is a dominant approach, however, which seems to have been preferred by countries including Romania, Austria, Denmark, Norway, Germany, and many others, who have adopted extreme social distancing measures going from case quarantine and public gatherings bans to partial lockdowns, closing schools and many workplaces, public transport, only allowing people to leave their homes for specific purposes, with an even tighter curfew imposed on the elderly. These measures were defended for their short-term capacity to reduce the rate of transmissibility and to flatten the epidemic curve as much as possible in order to primarily keep the hospital systems from getting overburdened.

The short-, medium- and long-term socioeconomic costs associated with these extreme measures are definitely a matter of discussion and have been putting pressure on countries to relax the situation. It has also been argued that “the incremental effect of adding another restrictive measure is only minimal and must be contrasted with the unintended negative effects that accompany it” (22). We actually begin to know more about some measures' effectiveness in containing the virus spread. For instance, combining case quarantine with other public health measures is shown to be more effective than only relying on case quarantine. When combined with contact tracing, the impact of some measures increases (23). Contact tracing combined with public disclosure of active cases' location seems to lower the number of deaths, having 50% lower economic costs than full lockdown (24). A comparatively cheap measure is to wear masks and some evidence suggests that wearing such can reduce transmissibility and be highly effective when compliance is high, at the same time substantially reducing both the death toll and the economic impact (25). Wearing them at 96% alone could flatten an epidemic growing at a rate of 0.3/day by bringing down the reproduction number from an original value of 3.68 to 1. But what about the other measures? How effective is it to close schools, close borders and to suspend or reduce national and international travels (26), or restrict certain workplaces' activity? And, finally, how much can a country build up its healthcare system during the restriction period?

A detailed analysis of all sectors of all countries is naturally a tremendous work and definitely beyond the scope of this article, and we will herein highlight some classes of measures for a more high-level perspective. There are various possibilities to combine measures in order to see their different effects in reducing the rate of transmissibility, while also looking at their different consequences under other criteria, including indirect deaths in different groups, inhibited work capacity in the longer and short term, or social costs, as well as their effects on democracy and human rights, among others.

We recognize that socioeconomic conditions, as well as healthcare capacities, can be very different, affecting the feasibility of some measures for particular regions, but also the quality of data. Therefore, any framework must be used with an awareness of national and regional conditions, and in health emergencies, the Global Health Security Index for instance can provide rapid data on a country's detection, response and healthcare capacity, as well as on its norms and risks so as to have a baseline when considering mitigation measures. The COVID-19 spread pattern also emphasizes that the model must be flexibly used and regionally adapted. Nevertheless, measures need to be based on adequate risk estimations of a situation as far as possible, including epidemiologic modeling and integrated analyses of the costs of reducing the risk, as well as a more systematic analysis of the extent to which various measures can reduce it. Because of the fluctuating data quality and other factors, any framework must be able to handle the various uncertainties involved. Furthermore, individual perception and factors influencing the said perceptions, including behaviors, narratives and framing, as well as the emotions stirred by media representations and by the level of uncertainty, must be taken into consideration during the deliberation. And there must be a preparedness which needs to be made in advance, as much as possible.

## The decision analytical framework

There are several studies investigating specific performance aspects of interventions against pandemics but they are most often limited to a single scenario and they are seldom designed to explicitly acknowledge the inherent uncertainties in both simulation results and scenario likelihoods. We have previously applied more dynamic multi-criteria decision analysis approaches to synthesize outcome predictions and stakeholder preferences from multiple perspectives into decision recommendations (27). Applied to the COVID-19 mitigation problem, the methodological components could, for instance, be partitioned into (i) a co-creative preference elicitation component, (ii) an epidemiological component, (iii) a socioeconomic component, and (iv) an aggregation and analysis component. The basic idea is to, relative to a set of possible mitigation measures, model the actual spread and its effects on the population with respect to critical health care, taking demographic and regional conditions into account, and furthermore estimate the effects from other perspectives, predominantly socioeconomic. A main point here is also that there should be adequate support tools for the deliberative process for structuring the decision situation and for providing information regarding possible measures and criteria. These processes should of course, to a large extent, be in place in advance and not conceived during an emergency, when there might be very little time for a more time-consuming decision apparatus.

### The Need for Participatory Components

The involvement of stakeholders in decision-making processes and model development is generally essential for catering to stakeholder requirements, but also for increasing the acceptability of the chosen set of measures. Policy-makers need to weigh their decisions against, among other things, the political costs of implementing sometimes unpopular sets of rules affecting social mobility, social interaction, or work organization. Not least in public health emergency situations, a distributed decision-making process could contribute to ensuring that the responsibility for the result is as well distributed, lowering the political costs and making way for a consideration of a variety of criteria relevant to the problem at hand.

A number of techniques may here be employed, relying on models from the decision-analytic field aimed at eliciting users' values through studying their preferences and gathering preferential data from several stakeholders in order to provide at least reasonable values, while keeping within the resource limits available (28). From the outset, it is usually a good idea to identify and have access to the relevant stakeholders for the problem which is addressed. In the case of situations such as the SARS-CoV-2 pandemic, aside from the first responders including the government, national institutes of public health and the sanitary system, social and economic agents should also be included since the non-pharmaceutical measures which are taken have a direct impact upon their activity. Among these, representatives of the business sector and in particular of the industries directly affected by the various measures discussed, such as the hospitality industry, retail, cultural, and educational sectors and transportation, should be part of the elicitation process. Of course, chain reactions affect other sectors as well including banking, suppliers and various small and medium enterprises which are affected by lowered consumption during various measures' implementations, so representatives of both business owners and employees would need to be included. Social groups need to be represented as broadly as possible through, for instance, relevant members of civil society with good knowledge, and experience with communities and municipalities. The need to protect vulnerable groups from the virus primarily concerns the care for the elderly and chronically ill patients, who are more exposed to serious forms of COVID-19. In addition to these, other groups who are directly affected by the measures under consideration include a variety of patients in need of healthcare whose access to medical services could be jeopardized during a lockdown, as well as women at risk of domestic violence, children and families at risk of poverty or precarious workers. Policymakers have the institutional legitimacy and capacity to call for broad participation in the elicitation process and many of them have had consultations with some of the stakeholders in order to, among other things, allocate supplementary funds and financial stimuli packages to mitigate the socioeconomic costs of a lockdown. However, such consultations are unstructured, often not transparent and can—intentionally or not—give a higher weight to some groups who are, for instance, more dominant or outspoken in the public sphere.

There are various guidelines to inform decision-makers of the acceptable norms that need to be taken into consideration when weighing the various policy solutions for managing the pandemic long-term, such as ensuring well-being, liberty and justice (29). This ethical component can be further detailed by including the ethics of care (30), where the moral salience of meeting the needs of vulnerable groups also implies the question of which vulnerable groups need more or equal protection in the current crisis, entailing perhaps equal weights for groups affected directly by COVID-19 and groups affected by the containment measures. It could be more informative (and perhaps less triggering in the public debate) to define the problem using cultural norms, drawing on cultural theories of risk (31, 32) which inform a criteria evaluation according to the analytic tool which distinguishes five cultural typologies—individualism, egalitarianism, hierarchism, fatalism and autonomy—characterizing people's preferences regarding how to manage, for instance, a pandemic. An individualist voice would choose a cost-benefit calculation, favoring a narrative that recognizes the trade-off between lives saved and economic costs, and between lives lost short-term and long-term. If the individualist would support a set of measures to “flatten the curve” as long as it would not bring intolerable economic costs [see, for instance, public statements voicing concerns that the cure must not be worse than the disease (33)], an egalitarian would reject economic considerations and cost-benefit analyses, placing a higher value on equity and on protecting vulnerable groups of the population first.

Depending on the available time frame, on the level of access to different stakeholder groups as well as on external circumstances which could make collaborative workshops difficult to organize (such as strict social distancing measures), various elicitation methods can be used to obtain rankings of the criteria with various degrees of robustness. Data from available surveys on social values (34–36) and cultural frames (32) can provide a preliminary hierarchy of people's values in the region where the measures have to be selected. Such frames can be identified in existing cultural analyses of specific regions, but they can also be extracted from public statements and texts circulated in mass media and social media, once the problem becomes part of the public agenda. These, however, have some limitations in eliciting evaluations from multiple stakeholders, as the visibility of different voices in the public sphere is not equal and is often affected by, among other things, restricted access, media partisanship, echo chambers, and institutional and commercial dominance. One of the challenges in designing a participatory approach to multi-criteria decision analysis is, therefore, to avoid reproducing the same inequalities in representation that are well-known in mainstream as well as social media.

A full societal analysis is far beyond the ambitions of this article, but it deserves to be emphasized that there are several options to create a transparent and deliberated framework for eliciting societal preferences. To demonstrate a comparatively uncomplicated method for at least obtaining a template for how a larger-scale survey could look, we designed a questionnaire by which to elicit some stakeholders' preferences (see Appendix 1) and tested it in Romania on a limited amount of stakeholders, addressing differences in risk perception and in assessing the severity of the risks. A continuation here could be the organization of stakeholder processes with the implementation of further engagement methods such as discussion workshops and forums when this is again possible vis-à-vis mobility and other restrictions. Our former experiences in particular regarding stakeholder workshops in a structured manner have been very promising (37).

### The Evaluation Process

A multitude of methods for analyzing and evaluating decision problems with multiple stakeholders and multiple criteria have been developed during the last decades. A fundamental component here is a set of criteria, under which the various options are considered. The possible measures to be taken are valued under each criterion and the relative importance of the criteria themselves is usually represented by a set of weights that can be defined in several ways. For instance, a set of criteria for the COVID-19 pandemic could include:

- Epidemiological & healthcare systems effects: (a1) direct fatalities, (a2) indirect fatalities;
- Economic aspects: (b1) short term costs, (b2) unemployment, (b3) taxes, (b4) specific industries affected, (b5) growing industries;
- Social and behavioral aspects: (c1) human rights, (c2) protection of vulnerable groups, (c3) criminality rates, (c3) mental health, (c4) education and training;
- Environmental: (d1) climate change;
- Political and governance: (e1) risk of short-term governmental abuse, (e2) citizen approval of measures, (e3) trust in the government, (e4) resilience – improving preparedness for catastrophic events;

as well as others, a set that can be refined after further literature reviews, projections and data elicitation from stakeholders. Our current set of criteria was established after media monitoring of pandemic response statements between February and June 2020, as well as after an initial round of research surveys of scientific and gray literature on COVID-19.

For the particular evaluations in our suggested framework, we use a method for integrated multi-attribute evaluation under risk, subject to incomplete, or imperfect information. The software originates from our earlier work on evaluating decision situations using imprecise utilities, probabilities, and weights, as well as qualitative estimates between these components derived from convex sets of weight, utility and probability measures. To avoid some mathematical aggregation problems when handling set membership functions and similar, we introduced higher-order distributions for better discrimination between the possible outcomes (38). For the decision structure, we use a common tree formalism. The data quality and regional conditions can be very different and there are thus large uncertainties in the background material that must be considered. We must therefore have a mechanism for taking this into account, but still being able to use the available data, even if the actual uncertainties are significant, and the use of, e.g., precise numbers is misleading. To alleviate some of the problems, we suggest a new evaluation method based on the resulting belief mass over the output intervals, but without trying to introduce further complicating aspects into the decision situation. During the process, we consider the entire range of values as the alternatives presented across all criteria as well as how plausible it is that an alternative outranked the remaining ones, and thus provide a robustness measure. Because of the complexity in these calculations, we use the software tool DecideIT for the analysis which allows for imprecision of the kinds that exist in this case. The tool is based on patented algorithms (39) and several versions have been successfully used in a variety of decision situations, such as large-scale energy planning (37), allocation planning (27), demining (40), financial risks (41), gold mining (42), and many others (43).

In the suggested framework, stakeholder preference elicitation is used for building preference structures where potential conflicts can arise. Here so-called surrogate weights have turned out to be useful, but since the elicitation can still be uncertain and the surrogate weights might not be a fully adequate representation of the preferences involved, we also work with intervals and their associated belief distributions, to accommodate for the uncertainties involved, cf. (38, 44).

The multi-criteria decision problem is evaluated as a multi-linear problem against the (imprecise) background information; in the next section, we provide the computational details of this process. Solving multi-linear optimization problems is generally hard. There have been several attempts to solve such problems, for instance, using active set methods or simplex-like methods using varieties of reduced gradients. There are also algorithms based on primal, dual, or primal-dual active set methods, that also are less suited for the problems that we have at hand. Further methods are based on linear complementarity programming theory, where iterative schemes are introduced. These general methods have their merits, but when working with imprecise information and using various kinds of sensitivity analyses, the decision problems that we are concerned with here become quite simple but non-linear indefinite. The main iteration of our particular method generates iterative sequences that are computationally demanding from an interactive point of view, why general methods are less adequate for such problems. We base the multi-linear solver on a set of algorithm libraries particularly designed for such problems (45). The details of these libraries are beyond the scope of this article. However, we below discuss the main principles from a conceptual viewpoint.

#### Rankings

We have in a number of papers argued for a set of alternatives to standard ways of addressing rankings in a computationally meaningful way. A promising such has turned out to be a new cardinal ranking method and we have there demonstrated that it is both more robust than the ones from the SMART family, AHP and many others, c.f., e.g., (46) for an overview. Below we briefly outline the main ideas behind this, using the notation from (47).

Assuming an ordering of *N* criteria, where we have an informal strength notation between the criteria as well as the measures in question, we suggest the translation:

>_0_ Equally important (as good as)
>_1_ Slightly more important (slightly better than)
>_2_ More important (better than)
>_3_ Much more important (much better than)

We use >_i_ to express the strength in the rankings between criteria and measures, where >_0_ is the usual ordinal ranking >. For instance, in a criteria ranking, we get a user ordering *w*_1_>_*i*_1__*w*_2_>_*i*_2__…>_*i*_*n*−1__*w*_*n*_. This is transformed into an ordering containing the symbols = and > by introducing auxiliary variables *x*_(*ki*)_:

(1) $$\begin{array}{l} w_{k}{>}_{0}w_{k+1}\;\text{is}\;w_{a}=w_{b}\\ w_{k}{>}_{1}w_{k+1}\;\text{is}\;w_{a}>w_{b}\\ w_{k}{>}_{2}w_{k+1}\;\text{is}\;w_{k}>x_{k(1)}>w_{k+1}\\ w_{k}{>}_{i}w_{k+1}\;\text{is}\;w_{k}>x_{k(1)}>\ldots >x_{k(i-1)}>w_{k+1} \end{array}$$

This establishes a new Euclidian space defined by the simplexes constrained by the new orderings and we obtain a computationally meaningful representation of the strengths. Now the number transformation of the criteria ranking is given by assigning a number to each position in the complete ordering, starting with the most important position as number 1. Each criterion *i* then get the position *p(i)* ∈ {1,…, *Q*}, where *Q* is the total number of positions. For every two adjacent criteria *c*_*i*_ and *c*_*i*+1_, whenever *c*_*i*_>_*s*_*i*__*c*_*i*+1_, *s*_*i*_ = | *p(i*+*1)* – *p(i)* |. Position *p(i)* thus represents the importance as stated by the decision-maker.

The weights are then obtained by

$$w_{i}^{CSR}=\frac{{}^{1}\text{​}{╱}_{p\left(i\right)}+\frac{Q+1-p\left(i\right)}{Q}}{\sum_{j=1}^{N}\left({}^{1}\text{​}{╱}_{p\left(j\right)}+\frac{Q+1-p\left(j\right)}{Q}\right)}$$

The transformation of the mitigation value orderings is analogous. In summary, the process is then simple:

1. For each criterion in turn, rank the alternatives from the worst to the best outcome. The strength is expressed in the notation with “>_I_” symbols.
2. For each criterion in turn, rank the importance of the criteria from the least to the most important. The strength is expressed in the notation with “>_I_” symbols.
3. The weighted overall value is calculated by multiplying the centroid of the weight simplex with the centroid of the alternative value simplex.

Thus, the transformation of the rankings does not introduce any computational difficulties.

#### Evaluation Method

What we actually evaluate here are special cases of expected values, weighted by criteria weights and (in some cases) probabilities. Furthermore, we use interval considerations that can be represented by random variables to take the inherent uncertainties into consideration. The general expected value in these contexts can be expressed as:

$$\begin{array}{l} E\left(M_{i}\right)=\sum_{i_{1}=1}^{n_{i_{0}}}w_{ii_{1}}\sum_{i_{2}=1}^{n_{i_{1}}}w_{ii_{1}i_{2}}\cdots \\ \;\sum_{i_{m-1}=1}^{n_{i_{m-2}}}p_{ii_{1\;}i_{2}}\cdots_{i_{m-2}{\;}_{i_{m-1}}}\sum_{i_{m}=1}^{n_{i_{m-1}}}p_{ii_{1}i_{2}}\cdots_{i_{m-2im-1i_{m}}}v_{ii_{1}i_{2}}\cdots_{i_{m-2im-1i_{m}}1} \end{array}$$

given the distributions over random variables *w, p*, and *v*.

We also introduce a belief calculus for evaluating structures, i.e., foremost a way of determining the beliefs in various parts of the weight and value intervals given interval input. This is to enhance the capacity to discriminate between the strategies and receive better estimates due to the possibility to use the information that the tree structure provides and the rapid concentration of belief mass as explained in details in (44). To evaluate this, we use the methods from (44), taking into account that there are only two operators of relevance here, multiplication and addition. The addition case is covered by ordinary convolution, i.e., assume that *h* is the distribution on a sum *z* = *x* + *y* associated with the distributions *f* (*x*) and *g*(*y*), then the resulting distribution *h*(*z*) is

$$\begin{array}{l} h\left(z\right)=\frac{d}{dz}\underset{0}{\overset{z}{\int }}f\left(x\right)\;g\;\left(z-x\right)\;dx. \end{array}$$

The multiplication case is quite similarly handled. With the same assumptions as above, the cumulative multiplied distribution *h*(z) is derived by first defining

$$\begin{array}{l} H\;(z)=\iint_{\Gamma_{x}}f\;(x)g\;\left(y\right)\;dxdy=\int_{0}^{1}\int_{0}^{z/x}f\;(x)\;g\;\left(y\right)\;dxdy\\ \;=\int_{z}^{1}f\;(x)G(z/x)\;dx \end{array}$$

where G is a primitive function to g, Γ_*z*_ = {(*x, y*) | *x*·*y* ≤ *z*}, and 0 ≤ *z* ≤ 1.

Then let *h(z)* be the corresponding density function:

$$\begin{array}{l} h\;(z)=\frac{d}{dz}\underset{z}{\overset{1}{\int }}f\;(x)\;G\;(z/x)\;dx=\underset{z}{\overset{1}{\int }}\frac{f\;(x)\;g\;(z/x)}{x}dx. \end{array}$$

Thus, the addition of the products is the standard convolution of two densities and the multiplication part is handled by a just slightly more complicated operation. Combining these two operations, we straightforwardly obtain the distribution over the expected utility.

The results of the process will then be a detailed analysis of each option's performance compared with the others, and a sensitivity analysis to assess the robustness of the result. During the process, the entire range of mitigation measures across all criteria can be analyzed as well as how plausible it was that a strategy would outrank the remaining ones, and thus provide a robustness measure for the stability of the respective strategies.

## An Example Application of the Framework

The following is an example of how plausible emergency responses can be systematically analyzed in a larger setting. We use our framework to evaluate different measures that could be adopted in Romania in response to the epidemic against a subset of criteria from the larger set described above in section The Evaluation Process. These were ranked by a small group of stakeholders in a consultation process using an online questionnaire which will be discussed in the next subsection. Then, we describe the input data to estimate the impacts of the alternative measures across every criterion.

Identifying the best set of measures to be implemented would firstly involve defining possible alternatives for Romania's response to the epidemic. Typical mitigation measures are partitioned into sets with different subordinate restriction levels, reflecting some important aspects of possible mitigation strategies, such as (14) going from an unmitigated epidemic to a suppression strategy or (48) proposing a schedule for every industrial sector activity in a risk adjustment strategy. Another option is to devise a set of measures that combines these approaches and also reflects the most common public debates on this issue:

- Level 1: An unmitigated epidemic—a scenario in which no other action is taken except pharmaceutical measures and case isolation;
- Level 2: Mitigation adding to pharmaceutical measures and case isolation, public communication encouraging increased hygiene and personal protection, localized action (closing a school/workplace in case of a number of cases)—influenza epidemics protocol
- Level 3: Mitigation adding to pharmaceutical measures and case isolation, personal protective measures (stay home when sick, hand-washing, respiratory etiquette, clean frequently touched surfaces daily, wearing face masks), mild social distancing measures (large public gatherings banned, work from home where possible, social distancing recommended);
- Level 4: Suppression (partial lockdown)—pharmaceutical measures and case isolation, personal protective measures (stay home when sick, hand-washing, respiratory etiquette, clean frequently touched surfaces daily, wearing face masks), imposed social distancing measures and restrictions on mobility: school closures, restaurants and large shopping centers closed, “stay-at-home” orders—as implemented in Romania for 2 months.

A full-scale multi-criteria decision analysis should also include collected data following a more extensive criteria setup which can be subject to refinement when gathering more available evidence, but for demonstrational purposes, we use the following criteria:

**Table:** 

**Health impact**
**• Direct fatalities**
**Economic impact**
**• Short-term costs**
**• Impact on specific industries**
**Socio-behavioural impact**
**• Human rights**
**• Vulnerable groups**
**• Access to education**
**• Mental health**
**Political and governance impact**
**• Risk of governmental abuses**
**• Resilience**

On these aspects, we have gathered stakeholder preferences as described below and we have estimated the values for the respective measures under each criterion, the input data being explained in sections The Choice of an Epidemiological Model and Socio-Economic Estimates. Needless to say, other data, such as business demographics data would be required to produce an estimate of how many lives can be saved as well as what the direct short-term and long-term costs of different risk mitigation measures would be. For our purposes here, we will handle this on a higher level of abstraction. Each component requires a significant amount of investigation in itself regarding the correlations between different factors, so the actual estimates herein are used for demonstration purposes only and can be updated with more extensive impact assessments. As more data becomes available from these fields, the model can be continuously updated for every criterion to produce new results, without its performance being affected.

### Eliciting Stakeholder Preferences

The participatory process was organized in the form of an in-depth web-based survey. The questionnaire for this survey was developed based on a comprehensive literature review about factors that are relevant for COVID-19 disaster risk reduction, and it addressed questions regarding risk perception, preferences for measures to be taken, and evaluations of relative criteria importance. We used an automatic web questionnaire (Appendix 1) to elicit stakeholder opinions, which was sent in June-July 2020 to 17 government officials, 16 healthcare experts, 11 representatives from the business sector, 9 non-governmental organizations, and 11 experts from academia. Sixteen respondents filled in the questionnaire, out of which three were medical doctors specialized in epidemiology, pulmonology, and public healthcare, five were university researchers specialized in sociology, political sciences, and philosophy, one was a representative of a workers' federation in Romania, and the rest were employees in the public sector and in NGOs. Since the purpose of the questionnaire was to both test the validity of the elicitation method for multi-criteria multi-stakeholder decision analyses on pandemic responses, and to obtain a sample of criteria rankings for our demonstrative evaluation, we consider the number to be sufficient, but not by any means representative at a national scale. In a full-scale setting, this should be quantitatively and qualitatively elaborated in a variety of respects, and augmented, e.g., via stakeholder discussion workshops, preferably supported by institutions with decision-making attributions in managing the epidemic crisis.

The result of this survey was that two-thirds of the respondents considered that an unacceptably high mortality of COVID-19 in Romania would have been between 1,001 and 5,000 deaths, a risk they considered to be very serious and very likely to happen^1^. One-third of the respondents (mainly sociology and public health policy experts) perceived the risk differently since the mortality caused by COVID-19 deemed by them to be unacceptable was significantly higher (between 10,001 and 20,000 deaths), an outcome which they estimated to be likely or very likely. Depending on this risk perception, stricter social distancing measures to keep the critical cases within the acceptable range can be justified or not, so a more representative number of respondents could ensure that the response to the current pandemic is not perceived as being disproportionate.

The most stringent problems brought by the SARS-CoV-2 pandemic in Romania were, according to the responses, the following (in no particular order): premature deaths and threats to people's health; the economic impact, including social and economic depression, loss of jobs, small companies closing down; the increased social isolation of the elderly and of those with less material means; overburdening the healthcare system, the lack of education for personal hygiene; the risks for mental health; the population's lack of trust long-term and disrespect toward rules, as well as the political calculations above medical and scientific interventions and the lack of evidence in decisions made. These suggested problems confirm the reliability of the proposed set of criteria for our integrated model of evaluation.

The survey asked respondents to evaluate 6 different measures, including alongside the ones we described a testing and contact tracing strategy and an enhanced isolation of the elderly strategy; however, since both of them are unfeasible for Romania, the former due to lack of infrastructural capacity and the latter due to a downright rejection of it by the public on ethical grounds, our evaluation focuses on the 4 alternative measures and selected criteria listed above. In evaluating the set of measures, respondents' preferences were expressed by ordering the given alternative measures, followed by the ordering of the different criteria and sub-criteria, from the least important (coded with the value 0) to the most important aspects (coded with value 14) for them. The results of their aggregated weights show that the measures preferred by respondents in mitigating the SAS-CoV-2 epidemic in Romania are the ones that have been applied in real-life (lockdown—Level 4 in our analysis), followed by the measures being applied during influenza epidemics (Level 2) and by Sweden's measures (Level 3). These weighed significantly more than not using any non-pharmaceutical measures to mitigate the epidemic (Level 1). In what concerns the criteria rankings relevant for our demonstration here, respondents considered that the health aspects were much more important than the economic impact, which in turn was seen as much more important than social and behavioral aspects. The political and governmental aspects were weighed as being less important than the social aspects. In what concerns sub-criteria, the impact on specific industries was considered more important than the short-term costs (measured here through GDP decrease). The impacts on human rights, on education and on mental health were seen as equally important, while the impact on vulnerable groups was considered much less important than the former aspects. How these weights are calculated in the formal decision analysis will be explained in section Measures and Criteria.

### Value Estimates

In this section, we will describe the data collection process on which the value estimates used in our impact assessments for the chosen criteria rely. Since the biggest degree of uncertainty, but also the justification of some countries' mitigation measures, resided in estimations of the virus spread and resulting fatalities, we will firstly present a model we have used to estimate the direct fatalities which would result in the 4 different scenarios under evaluation. Secondly, we will describe socio-economic data on which our assessments were based. What is important for the evaluation of alternatives is to have variables that indicate the impact of every set of measures at a local level; thus, matters such as infrastructure (number of hospitals, ICU beds, or ventilators), access to healthcare or institutional capacity, which are not influenced by the non-pharmaceutical measures considered here, represent the local benchmark used when comparing the impact estimations. Such a benchmark could be set, as abovementioned, by the Global Health Security Index (1) or by the INFORM Risk Index (49), where Romania's estimated risk class is low, but its institutional coping capacity is at high risk (INFORM Institutional 5.7).

#### The Choice of an Epidemiological Model

In epidemiological modeling, there are various tools available for supporting scenario analyses and (assumingly) producing acceptable forecasts in a timely manner. System Dynamics is a natural choice for implementing models simulating transmission processes since the methodology presupposes a holistic approach and focuses on how the parts in the system affect each other with reinforcing or balancing feedback loops (50, 51). The family of SEIR (Susceptible, Exposed, Infected, Recovered) models are quite common to represent the spread of disease in a population, where people are divided into compartments depending on their immunity status. In these models, a system of coupled differential equations governs the flows between the different compartments over time, where people becoming infected move from S to I and people who recover (or die) move from I to R. SEIR models usually operate on individual mortality, disease spread rate, recovery rate and the mean infection time, rate of movement from the exposed class to the infectious class, the mean latency period, and the basic reproduction number R_0_ (52). During the latter decade, various simulation environments have also emerged, such as AnyLogic, enabling for swift usage of, e.g., generic SEIR modeling which has been used in some recent studies, including studies of the Corona SARS-2, MERS, and the Zika virus (53, 54). Alternative models are taking more parameters into account and hopefully producing better predictions. For instance, in Sweden, the National Board of Health and Welfare has supported research and development of a decision support tool to complement the individual-based, total population model MicroSim (55, 56). In order to model the effects of containment measures applied for a specific demographic, models such as (57) or the COVID-19 scenarios at the University of Basel (58), or another candidate in the abundance thereof, could also be considered depending on the circumstances and the available level of specificity for the data sets, social characteristics and healthcare capacity. There are still many critical uncertainties with COVID-19 and every model with higher complexity than the training and validation data should be used very carefully as a decision basis, in particular since SARS-CoV-2 does not seem to behave like, e.g., a seasonal influenza, but is instead acting more “local,” why the micro and meso perspectives must play an important role.

For our purposes herein, we apply regionalized demography augmented SEIR model for modeling the health effects of various risk mitigation measures. The model thus requires country-specific information (see Appendix 2) including population size in country/region/city divided into age groups, so as to model the effects of various measures in the desired area. It moreover can include morbidities in the population per age group, in as much as these figures are available in national statistics and relevant literature, and current numbers of confirmed cases per day, divided per age group and case severity. The benchmark for the medical system capacity (no. of ICU beds, ventilators, medication, testing capacity) should in principle be run against structural possibilities to increase it in a given timeframe, namely access to national or international funds, workforce capacity, relevant research, etc. As will be seen from the example demonstration below, it seems to work quite decently, but can be substituted by any other adequate one if preferred and more data is available.

The simulations of the measures' effects in containing the virus spread in Romania below were made in AnyLogic 8, based on a data set that should be adjusted and adapted to different regions. As input, the model uses the Romanian population divided into three age groups: 0-19, 20-59, and 60 years or older, according to national severity profiles which show a higher incidence of severe COVID-19 cases and deaths in the 60+ age group, due to existing comorbidities (59). The number of days between being infected to becoming infectious is, on average, 5.1 days (60, 61), and the time being infectious 5.0 (62). The model was fitted against the daily number of reported cases, fatalities and ICU occupancy in Romania by January 3rd, 2021. An infectivity parameter, a relative contact reduction, and the proportion of unreported cases were calibrated for each age-group. Unreported cases were assumed to be less infectious than reported cases, considering that these have milder symptoms. The contact profile changes three times during the simulation, and we have two periods with different infectivity and share of unreported. Further details regarding input parameters are found in Appendix 2. This baseline scenario was then used to simulate the various strategies of mitigation, starting with January 3rd, 2021. The 14-day case notification rate per 100.000 was 253.08, a significantly increased rate compared to the June-July period when the rate was 31.2.

**Figure 1:**
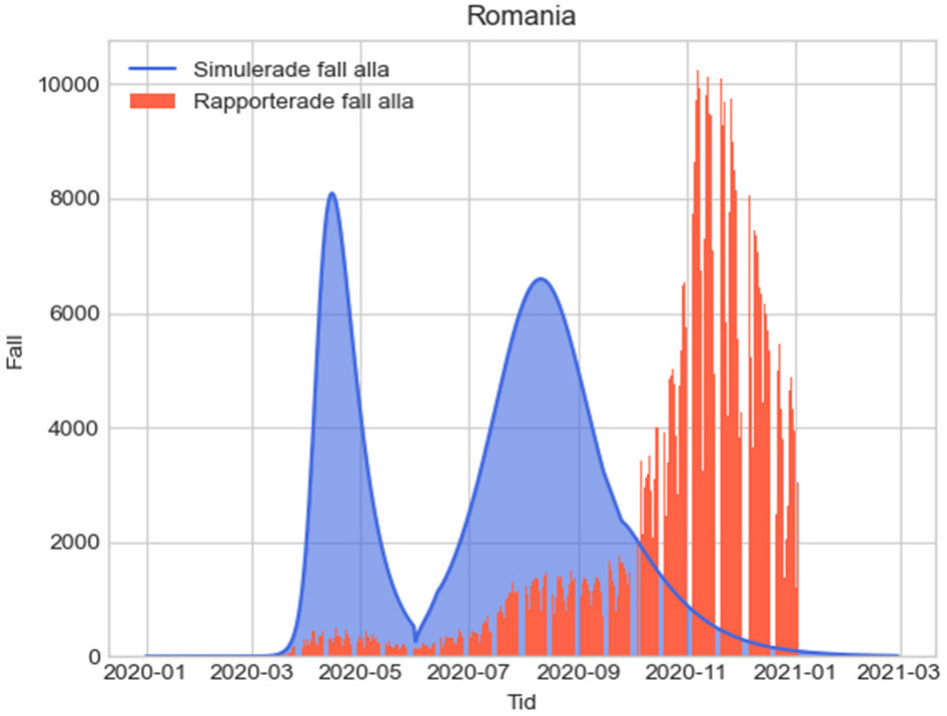
L1: Epidemiologic evolution without any social distancing measures. Total unreported cases 2020-2021: 14,260,483.4; total unreported cases 2020: 14,260,483.4; total estimated fatalities 2020: 32,799.11182.

**Figure 2:**
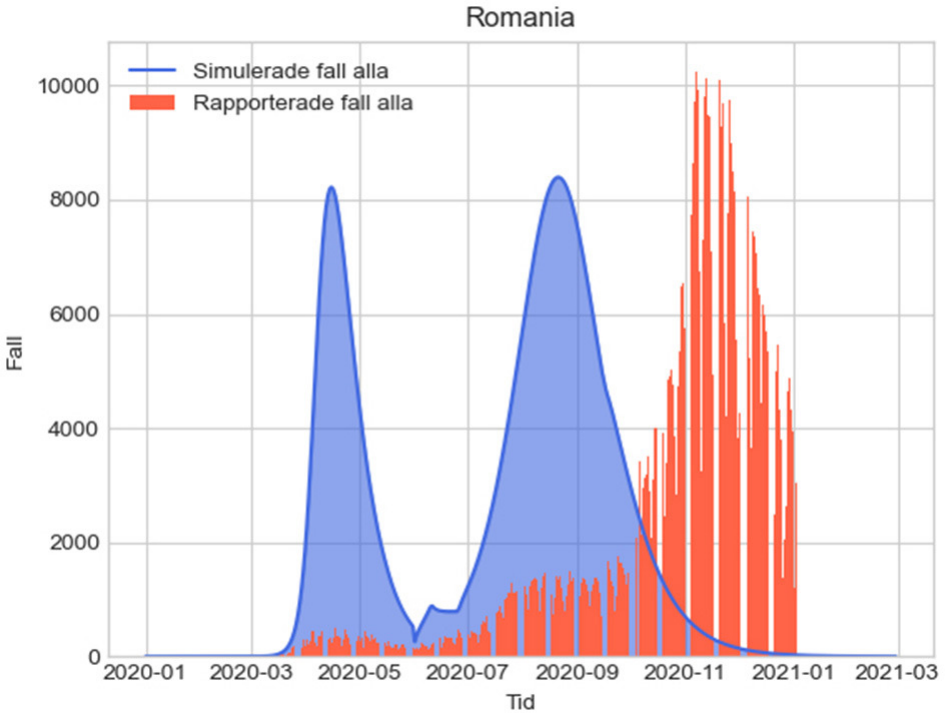
L2: Epidemiologic evolution using the influenza season protocols. Total unreported cases 2020-2021:14,855,222.113; total unreported cases 2020: 14,847,477.491; total estimated fatalities 2020: 34,149.19.

**Figure 3:**
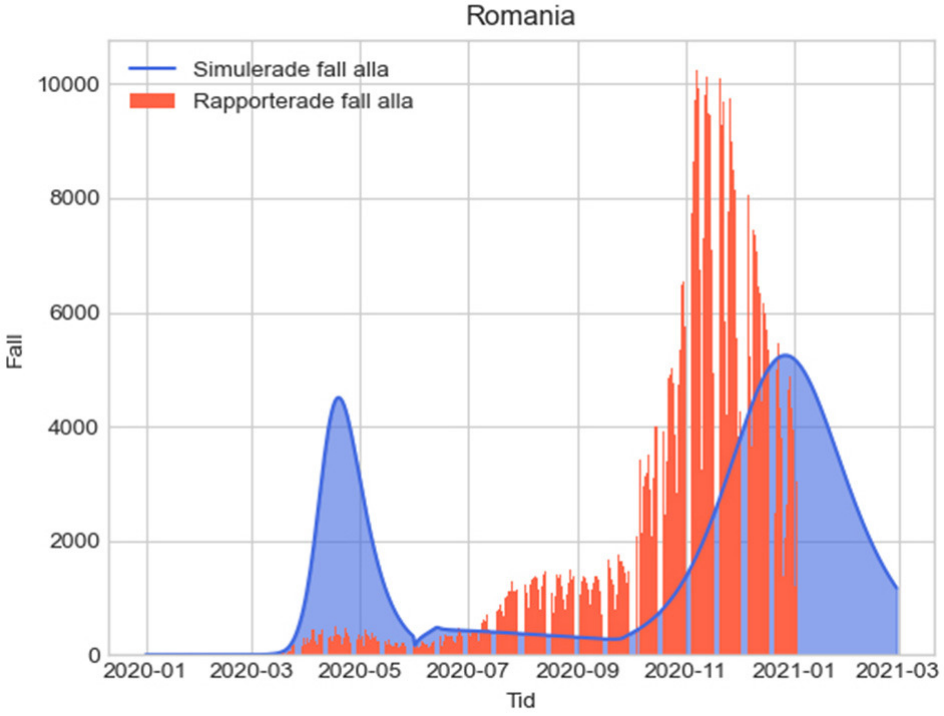
L3: Epidemiologic evolution with social distancing recommended. Total unreported cases 2020-2021: 12,930,507.92; total unreported cases 2020: 9,623,212.942; total estimated fatalities 2020: 22,133.38.

**Figure 4:**
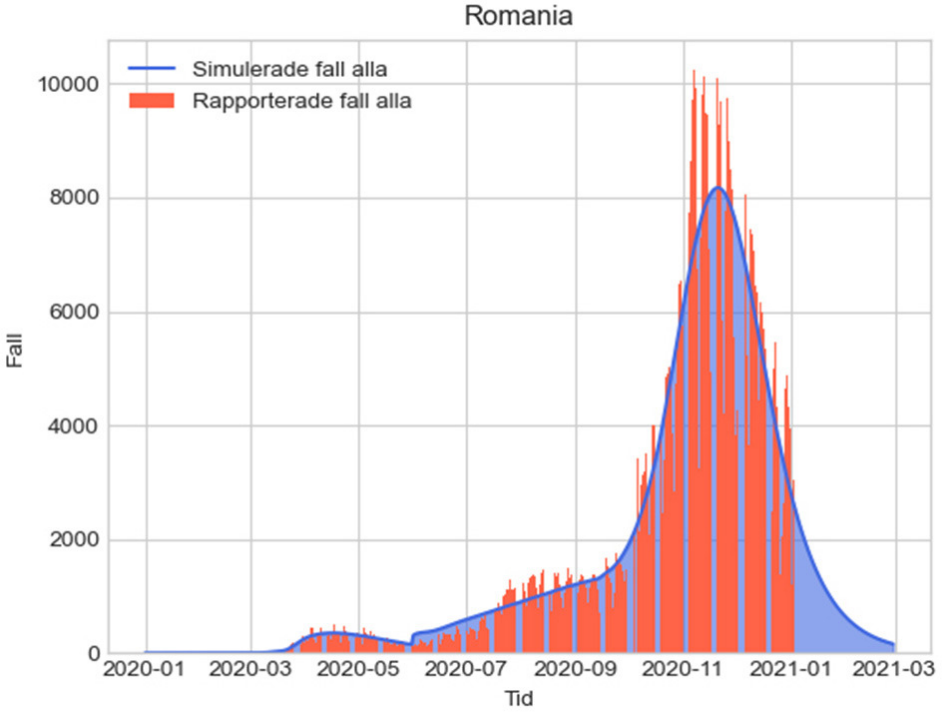
L4: Epidemiologic evolution with suppression for 2 months. Total unreported cases 2020-2021: 13,490,259.84; total unreported cases 2020: 12,525,884.439; total estimated fatalities 2020: 28,809.53.

The results from the four alternative measures with their assumptions are provided in Figures 1–4 below, where the simulated results from December 31, 2019, to the end of 2021 are shown together with the actual reported cases by January 3rd, 2021. Since our example uses values estimating the impact of various measures for the year 2020, in estimating direct fatalities we have summed the total of unreported infections in one year for scenarios 1-4 and then assumed an infection fatality rate of 0.23 (63), which can of course be modified accordingly when other values are established.

In the figures below, the red graphs show the number of simulated reported positive cases per day, and the blue ones show the simulated unreported cases per day:

Since the estimations do not take into consideration other factors such as improvements of treatments, regional patterns of spread and other variables, we used a 10% confidence interval for the total estimated fatalities. In this case, in scenario 4 using the real-life measures taken in Romania, we obtain an interval of [25929; 31691]. This is consistent with the sum of reported fatalities in Romania caused by COVID-19 in 2020 (around 17,000) and excess deaths caused by conditions that could reasonably be attributed to undetected COVID-19, such as among others, circulatory system diseases (10,000 more deaths than in 2019). Recent analyses based on excess mortality in Romania in 2020 also suggest that the real figure of COVID-19 fatalities was most likely over 26,000 in the first year of the pandemic (64).

#### Socio-Economic Estimates

If the number of total COVID-19 fatalities for every level was obtained using the abovementioned epidemiologic model, the estimated socio-economic impact of every set of measures was based on various data sources and indices, which will be explained below. These estimations can, of course, be refined at any point. The framework does not depend on the input data or on certain epidemiologic or economic models chosen to generate such data. However, the results of the final evaluation of alternatives do depend on the input data, therefore the following evaluation is subject to change if different data will be produced. Note that in the evaluations, we make the quite uncontroversial assumptions that more cases, less GDP decrease, and fewer students getting education are considered to be inferior to fewer cases and so on.

The short-term costs are measured by GDP growth, which was−5.0 in Romania in 2020 (65), this corresponding to our Level 4, the real-life scenario where the two months of lockdown included closures of non-essential shops, restaurants, theaters and schools, among others. Employees working in affected sectors were sent to technical unemployment, and the government introduced a deferral of payment of certain taxes and utilities, as well as a moratorium on loan repayment for companies and individuals. Monthly estimates of GDP growth in 2020 recorded by the COVID-19—Romanian Economic Impact Monitor (66) show that the GDP growth forecast was estimated at−10.3% during the lockdown, followed by−5.7% in July-September and by−1.5% in October-December 2020, as various sectors were allowed to reopen their activity and citizen mobility increased. Taking into consideration the various trans-border effects of measures taken within the EU and globally, affecting macroeconomic indicators and some sectors' activities, including trade and tourism, we have estimated GDP deficits for other scenarios as being slightly smaller in case of recommended social distancing and much smaller in case no social distancing measures are introduced. Similarly, the effect of the four different mitigation strategies upon specific sectors' economic activity gradually worsens as more sectors are either closed or are indirectly affected by closures and imposed social distancing. According to the abovementioned Economic Impact Monitor, economic activity indicators show that, aside from health services and the public administration sectors, all other economic sectors were negatively impacted, the most affected industries being tourism and hospitality (-64.4% in Q2 of 2020), culture and arts (-60.4%), and the heavy industries (-29.1%). In Q3, corresponding to a Level 3 stringency level in our evaluation, most sectors recovered, aided by governmental fiscal facilities as well, except for agriculture (-19.4%). It is, thus, reasonable to consider that the more stringent the measures are, the more industries get negatively impacted, resulting in the ordinal ranking in Table 1 below.

**Table 1:** The value estimates for the respective measure under each criterion.

**Criterion/Measure**	**Health**	**Economic**		**Social and behavioral**				**Political and governance**	
	**Direct fatalities**	**Short term costs**	**Impact on specific industries**	**Human rights**	**Vulnerable groups**	**Access to education**	**Mental health**	**Risk of abuses**	**Resilience**
Level 1	29438.1–35979.9	1-3	Better than L2	Better than L2	1.4	0	Better than L2	6.49	47.9
Level 2	30733.2–37562.8	1-4	Better than L3	Better than L3	1.4	14-28	Better than L3	6.49	44.9
Level 3	19752.3–24141.7	3-5	Much better than L4	Better than L4	1.6	0	Better than L4	6.44	50.9
Level 4	25928.6–31690.5	5-6			1.7	54-84		6.4	41.9

Qualitative assessments are also made for two socio-behavioral criteria, namely human rights and mental health. The impact of alternative measures upon these aspects also worsens as stringency levels increase; before introducing lockdown, the Romanian state activated Art. 15 of the European Convention on Human Rights on March 15, 2020. The derogation gave the government broad powers in taking measures to contain the spread of the virus, trading off rights such as access to healthcare, freedom of movement, freedom of assembly, access to justice and access to education (67). For two months, both public and private hospitals suspended healthcare for all non-emergent medical cases by a governmental order, affecting chronic patients' treatments: compared with 2019, in April and May 2020 there were 70.98% and, respectively, 61.48% fewer hospitalizations, and specifically around 80% less chronic patients' hospitalizations (68). In Romania, there are 17,500 TB patients, 16,500 HIV patients, over 1 million diabetes patients and 488,824 cancer patients. For estimating the impact on human rights of other measures, we take into consideration border restrictions, case quarantine and temporary school closures (Level 2), as well as limits to the freedom of assembly through bans of large gatherings (Level 3).

In what concerns mental health, preliminary reports from the COH-FIT project (69) on Romanians' mental health during the pandemic show worsening stress and nervousness levels reported by almost half of respondents within the population aged 28-50 years old, as well as an intensification of pre-existing conditions reported by a third of respondents, and an increased sense of loneliness. The reported factors which exacerbated the impact were poverty, unemployment, physical diseases and the loss of a loved one. During lockdown, a series of five national surveys on Romanians' perceptions, attitudes and behaviors, conducted by the Romanian Institute for Evaluation and Strategy (IRES), showed that loneliness was substantially reported by teenagers and by the elderly respondents, while 4 in 10 respondents reported they feared losing their means of livelihood because of the crisis (70). Separating measures' effects from the effects the pandemic itself is difficult since COVID-19 and the fear of disease can cause declining mental health and well-being on their own, as reports have shown. However, declining mental health due to isolation and financial scarcity associated with job losses can be attributed to mitigation measures. Therefore, we have considered that the impact of Levels 1 and 2 on mental health is smaller than the impact of Level 3, which involves social distancing, the highest negative impact on this criterion being under Level 4.

For estimating the impact of various measures on education, we looked at the number of school days lost in each case. During the lockdown period, an initial school closure for 18 days led 3,526,200 students to not have access to education. After this, the educational activities were resumed online for another 36 days, but an estimated number of 903,870 students (32% of pre-university students) did not have access to distance learning (71) due to lack of material means, such as digital devices or internet access. From September until November 2020, more localized measures were introduced, whereby the choice for face-to-face, hybrid or distance learning was continuously revised based on incidence rates at county levels, therefore a precise number of days lost during this period is difficult to estimate. After November 9th, all schools switched to distance learning. In estimating the number of days for other measures, we assume schools do not close (Levels 1 and 3) or that only schools with 3 confirmed cases switch to distance learning for 14 days (Level 2).

In estimating the impact of mitigation measures upon vulnerable groups we used values from the INFORM Index for Risk Management (49), where Romania had a score of 1.7 for the Vulnerability component for 2020. Compared to 2019, this score has remained constant and the Vulnerable groups indicator has slightly improved (from 1.5 to 1.4), but data reliability in estimating its sub-indicators could be affected by the reduced access to healthcare during and after lockdown by chronic patients, as described above. Moreover, the socio-economic vulnerability has worsened, in particular with regard to inequality (from 2.7 to 3.5). For these reasons, we use the general Vulnerability Index in our estimates, which we suggest would be lower for Levels 1-3, in correlation with less severe economic impacts.

Finally, we have used two more indices, this time to assess the measures' effects on political and governance aspects; the risk of governmental abuses was measured through the 2020 Democracy Index (72) and the impact on resilience was estimated using Bloomberg's Covid Resilience Ranking (73). Compared to 2019, the functioning of government has slightly worsened (from 5.71 to 5.36), decreases in political culture (from 4.38 to 3.75) and civil liberties (from 7.65 to 7.06) also being noticeable during the pandemic. We assume that, as civil liberties would increase under Levels 3, 2, and 1, so would Romania's democracy score. Other sources can of course be used, such as the Political and security risk and the Socio-economic resilience indicators of the Global Health Security (GHS) Index, both indicators mainly relying on data from The Economist Democracy Index.

Needless to say, the values should, in an extended analysis, be refined through economic models, empirical data, more well-deliberated qualified estimates, etc. The measures considered under the respective criteria are summarized in Table 1.

### Measures and Criteria

As described in section Eliciting Stakeholder Preferences, the sampled opinions are too few to be representative and the example questionnaire is not granulated enough for a real model input, which is why the limited representation below should be considered as a model demonstration and not a policy recommendation. It nevertheless indicates that this representation format actually is very feasible and should be quite straightforward to use in an extended study, thus we suggest a representation of a subset of the preferences as a ranking of the criteria:

CH2: Direct fatalities >> Economic >> Social and behavioral > Political and governmental
CH1: Industrial effects > Short term GDP
CH3: Human rights = Access to education = Mental health >> Vulnerable groups
CH4: Resilience >> Risk of governmental abuses

**Figure 5:**
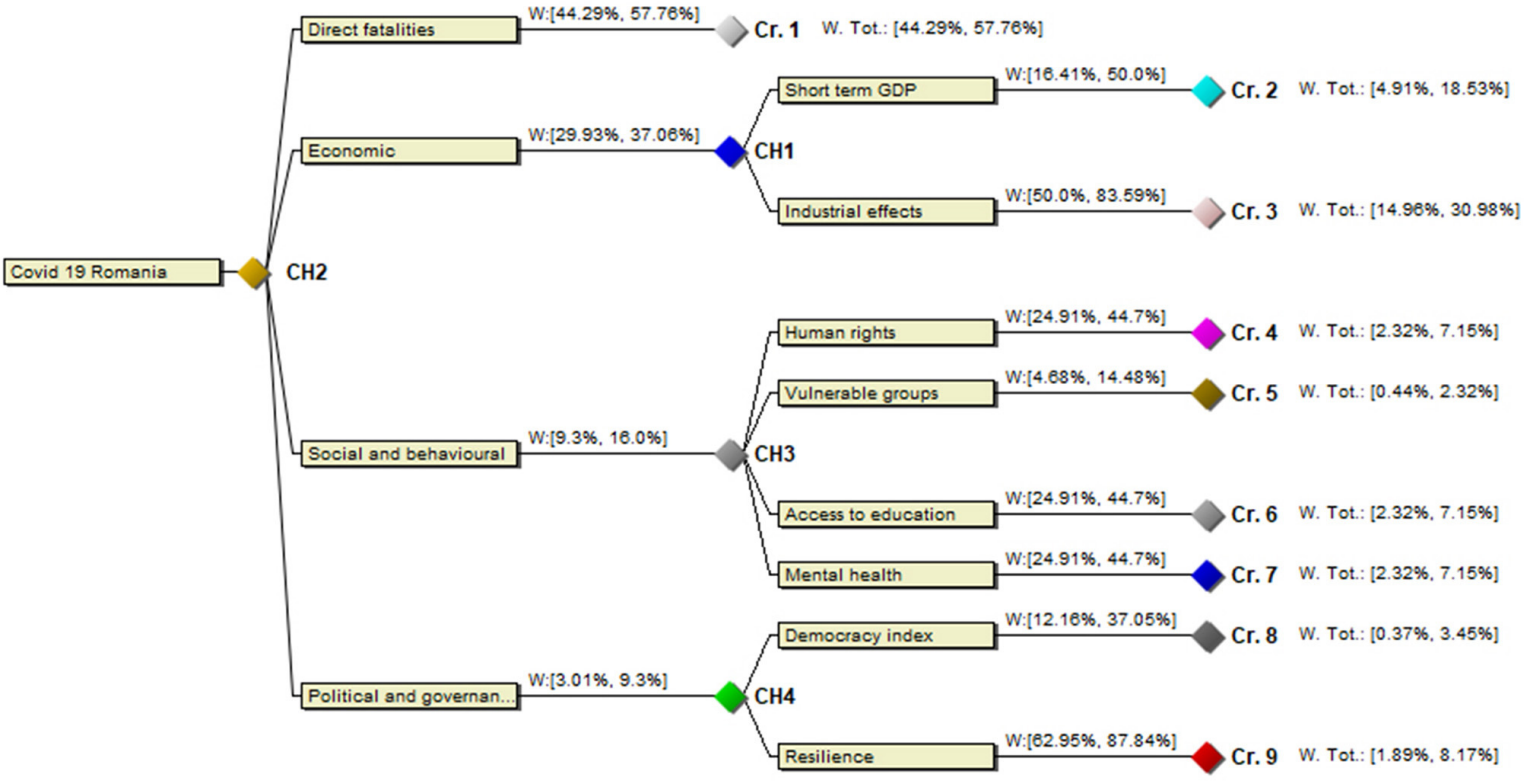
Criteria tree for COVID-19 response measures in Romania.

This is not a pure ordinal ranking and we have to use a different representation thereof. We need supplementary statements for the criteria to calibrate the different scales involved since they are of very different character and we simply assume (because a formal P-SWING procedure was not performed) that this representation becomes the criteria tree in Figure 5.

We then again use the notation from (37) to represent the strength of the rankings between the criteria by introducing auxiliary variables x_i_ and we obtain the ranking w(fatalities) > x_1_ > w(economy) > x_2_ > w(social) > x_3_ > w(political), denoting the weight of fatalities by w(fatalities) and so on. This theory behind the process is explained in detail in ibid. Using the more elaborated theory, we could considerably have refined the elicitation of the rankings between criteria, but such an analysis is beyond the scope of this article. Finally, for the alternatives, we have a mixture of interval estimates and a ranking.

### Aggregation and Evaluation

**Figure 6:**
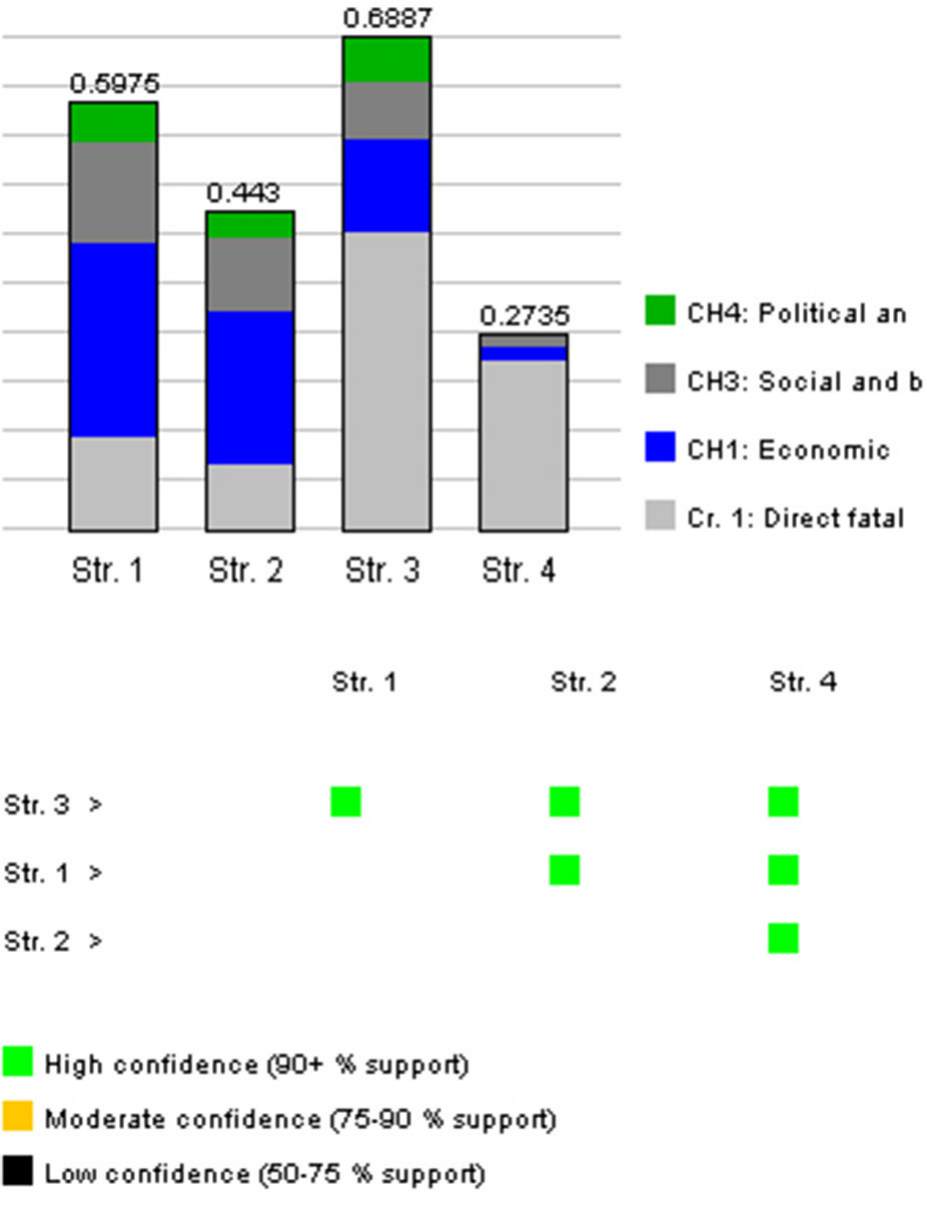
The result of the DecideIT evaluation showing ranking, the criteria contribution as well as the significance of the result.

The multi-criteria decision problem is evaluated against the background information using the method described in section Evaluation Method above. This means in this simple case, without sub-criteria, that we evaluate weighted averages of the figures involved, or, more precisely, equations of the format E(M_j_) = Σ w_i_v_ij_, where w_i_ is the weight variable of criterion *i* and v_ij_ is the value variable of measure *j* under criterion *i*. The value E(M_j_) is computed by solving successive optimization problems by the program DecideIT, implementing the ideas described in section Evaluation Method. The result of our example is provided in Figure 6.

In the figure, the higher the bar for the measure, the better it is, given the background information. The bars also show how much each criterion contributes to the respective values, based on the possible ranges of the resulting weighted averages of the respective measures. Furthermore, the robustness of the opinions is color marked. Green means that there is a significant difference between the features and that there must be substantial changes in the input data for it to change. Yellow means that there is still a difference, but it is more sensitive to input data. Black means that there is no significant difference between the desirability of the measure. The confidence measure just the proportion of the volume under the resulting distribution as explained in section Evaluation Method. An extended explanation of the semantics regarding the bars and the color markings is also provided in (28).

In summary, the differences are all significant where L3 is the best strategy, followed by L1, L2, and L4. L3 is clearly the best option in this example. Furthermore, this result is quite robust. We can also note how this significantly differs from the uninformed intuitive rankings from the results of the questionnaire.

**Figure 7:**
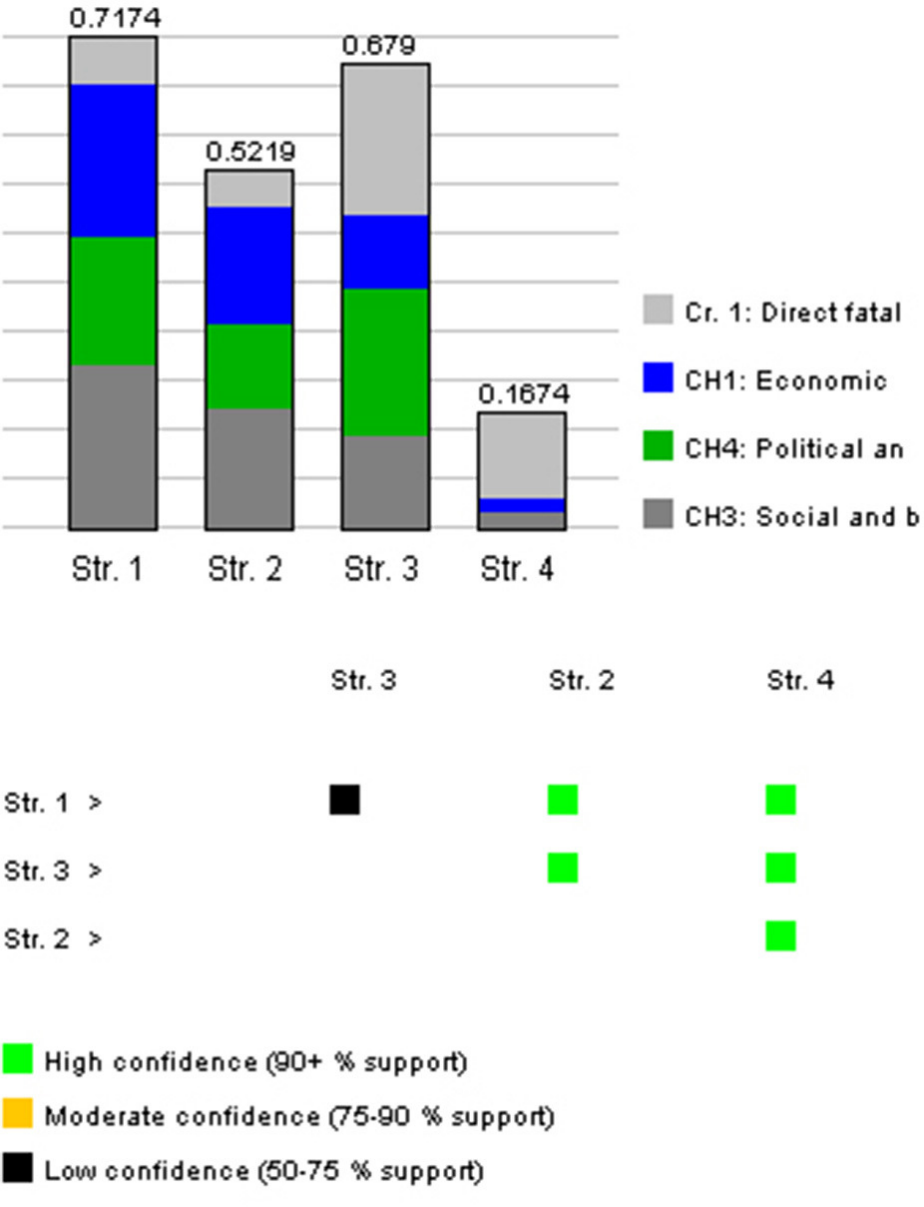
The result of the DecideIT evaluation when all main criteria are unweighted. Conclusion: “Str. 4: L4” is the best strategy, with “Str. 1: L1” in second place. The statement Str. 4 > Str. 1 is made with low confidence since the information provided in the model supports this to a degree of 60%.

Needless to say, different data would affect the result. For instance, if we consider when all main criteria are unweighted, given the value ranges, the result would be the one in Figure 7. As can be seen from the figure, the ranking is changed, but the difference between L1 and L3 has lower confidence.

Note again that our point here is not that this in any way provides a conclusive recommendation plan. Our purpose here is to demonstrate a methodology for solving such complex problems under large uncertainties in multi-stakeholder settings and to show as well that there are effective tools available for the quite elaborate calculations involved.

## Concluding Remarks

In this paper, we have presented a policy- and decision-support framework for managing the response to the SARS-CoV-2 pandemic and other future hazard scenarios, characterized by a large degree of uncertainty. The framework can be implemented both during emergency preparedness and ongoing response, by relevant authorities and experts alike. Naturally, the more reliable data on relevant criteria, the better, to obtain evaluation results that have a higher degree of confidence. However, without an adequate decision mechanism to aggregate and evaluate data, and without a stakeholder consultation process to establish the local priorities in mitigation response, epidemiologic data alone cannot automatically translate into appropriate policies. We thus recommend policy-makers at national and regional levels to use multi-criteria decision support tools and multi-stakeholder frameworks in deliberating upon the best course of action in current and future hazard scenarios. The framework should be regionally adapted and used, given differing socioeconomic conditions across a state, as well as different spread patterns. This is why the stakeholder consultation component is meaningful since sociocultural groups can have different priorities for particular regions. Obtaining regional socioeconomic data can pose some difficulties as it depends, among other things, on reporting protocols and on chain effects with other regions. However, the set of criteria employed can be tailored to the needs and capabilities of any region.

Crisis scenarios are indeed tremendously complex from a societal viewpoint and can result in highly undesirable side effects as well, which is why an approach cannot be restricted to a single criterion, such as fatality rate or financial short-term effects, but should rather be situated within a wider field of social shaping. There is certainly a multitude of relevant aspects on the current crisis and the main purpose of this article has been to suggest what a framework for pandemic modeling, including epidemiological and socioeconomic factors, could look like, as well as to emphasize that such analyses should really be done as a basis for evidence-based policymaking regarding pandemic situations. Representing complex scenarios in socioeconomic systems has the potential to inform policy formation processes, and we believe that such a framework can decrease irrational decisions disturbed by a variety of cognitive and political biases as well as reducing the number of measures with insignificant effects or with highly undesirable side-effects.

The transformation of societal systems cannot be determined solely by any technological or economic assumed rationality. Rather, there is a wide range of social, political and institutional factors that interact in a systemic fashion and influencing their development. The acknowledgment of the multiple factors at stake in handling the crisis has more often than not been omitted from public communication, where public officials' statements mostly framed the problem unilaterally, basing their narratives on warnings coming from the medical and public health scientific community. Since the current pandemic has primarily been considered a public health problem, strategies to mitigate the direct impact of COVID-19 upon the population have been persuasively communicated. Ethically justifiable use of narratives in science and evidence communication should, in principle, act for the common benefit and not “restrict an individual's autonomy to make decisions” (74). Persuasion can be used where there is a high consensus that science “can justify the best course of action,” in particular for emergency actions. However, the assumed best course of action must be carefully deliberated and motivated.

Our study provides a feasible methodology for structuring available – even if imprecise – evidence and preferences, which also serve as a support for publicly communicating the decision-making process. The long-term effects require sub-decisions as well, further complicating a naturally simplified analysis. For instance, macroeconomic policy actions and fiscal measures are critical to longer-term effects, something that the various types of austerity measures in the aftermath of the global financial crisis have emphatically highlighted, as well as to their effects on other criteria involved such as mental health (75, 76) and the irrational growth of political populism and power abuse as well as distractions, cf. (77–79). Furthermore, international comparisons are problematic due to the regional nature, as well as other factors, of the COVID-19 spread patterns. Therefore, comparisons between national strategies are very difficult to evaluate in a reasonable way. For instance, Sweden as a quite interesting case has taken a different approach compared to many other countries, but the result could have been very different in countries with different healthcare systems, demographics, telecommunication situations, authority trust and relations to social contracts, traveling patterns and so on. Therefore, a framework like this must be used with an awareness of national and regional conditions. The COVID-19 spread pattern furthermore emphasizes that the model must be flexibly used and regionally adapted.

It is also difficult to adequately make trade-offs between different criteria, in particular when the stakes in many cases are high, but trade-offs must nevertheless be considered when handling such situations and it should be transparent which they are and how they affect the actual decision making, even if the trade-offs are not always clear (80). As it now happens, these are often hidden, making it impossible to scrutinize the decisions that have been taken. For instance, the 70+ age group accounts for an overwhelming number of all deaths. Areas, even in reasonably wealthy countries or regions, having a higher proportion of first- and second-generation immigrants have been significantly more affected. How should this be considered compared to other effects? Should there be another type of precautionary measures and even society constructs so that particularly vulnerable and socially underprivileged groups are better protected when these types of events occur? In an international setting, such questions will be even more important in a variety of respects, not the least since many countries will suffer tremendously from the various socioeconomic side-effects of pandemics, exacerbating poverty and inequality, even aside from the much higher direct effects due to limited health care systems. These kinds of questions must nevertheless be clarified in advance and well-anchored in the broader populations, another reason why transparent and deliberated policies should be analyzed and in place beforehand. To do this, there is a need for integrated methodologies and decision processes for how country strategies and action plans should be aligned with overall objectives and stakeholder perceptions and preferences. Deliberated strategies must be a prerequisite for policy formation and they should furthermore be developed together with the civil society in order to be better prepared for future crises.

In a deliberate design, stakeholders would be made more aware of the availability of different options regarding each of the pertinent hazards to their communities, as well as the impact of their preferences on risk management and on the broader society. This would probably facilitate improvements in resilience as well to future extreme hazard events, particularly in a multi-hazard context where it could deliver effective solutions for a multi-stakeholder planning approach and strengthen policy coherence by identifying management options, thereby contributing to more resilient regions. The management options can be communicated with stakeholders who could also be used to gather feedback about how they recognize these options and determine the possible opportunities and constraints from their viewpoint. The participatory approach of engaging different stakeholders would help to ensure the buy-in of stakeholders and encourage them to take on board the final results and raise the understanding for various measures, while still being aware of side-effects that are violating other fundamental societal effects. If this work could be undertaken, an applied framework would then define a blueprint for how crisis preparedness could be better carried out, implemented and scaled up.

## Data Availability Statement

The raw data supporting the conclusions of this article will be made available by the authors, without undue reservation.

## Author Contributions

All authors listed have made a substantial, direct and intellectual contribution to the work, and approved it for publication.

## Conflict of Interest

The authors declare that the research was conducted in the absence of any commercial or financial relationships that could be construed as a potential conflict of interest. The reviewer P-EM declared a shared affiliation, with no collaboration, with several of the authors LE, TF, and MD to the handling editor at the time of the review.

## Footnotes

^1^When the questionnaire was sent, there had already been over 1,500 deaths caused by COVID-19 in Romania.
