# Supplementary material for: A Multi-Criteria Framework for Pandemic Response Measures
Source: Front Public Health. 2021 Apr 20;9:583706. doi: 10.3389/fpubh.2021.583706 (PMC8096778; doi:10.3389/fpubh.2021.583706)
Supplement: Supplementary file 1 [file Data_Sheet_1.pdf]

Appendix 1 - Stakeholder questionnaire: Risk perceptions and preferences in mitigating the SARS-CoV-2 epidemic in Romania

Expertize / Field of work

Sector/industry: Government / Institute for public health / Medical facility / Public sector / Private sector / Academia / Non-governmental sector

1. For Romania, what would you consider to be an unacceptably high mortality caused by COVID-19?

☐1,001-5,000	☐5,001-10,000	☐10,001-20,000	☐20,001-50,000	☐50,001-100,000	☐100,001-1,000,000	☐over 1,000,000

2. In your view, how serious is the risk of high mortality rates caused by COVID-19?

☐very serious	☐serious	☐somewhat serious	☐not very serious	☐not serious at all

3. How likely do you think it is for the SARS-CoV-2 epidemic in Romania to have a high mortality rate, if left unmitigated?

☐very likely	☐likely	☐somewhat likely	☐not very likely	☐unlikely

4. What is, in your view, the most pressing problem in Romania brought by the SARS-CoV-2 pandemic?

5. In your view, which of the following non-pharmaceutical measures to contain the SARS-CoV-2 epidemic in Romania would be better? Drag the slider scale using the mouse to indicate the order of preferences.

- No other action is taken except for pharmaceutical measures and case isolation;
- Health system coordination, public communication encouraging increased hygiene and personal protection, case isolation and localized action (closing a school/workplace in case of a number of cases) - *influenza protocol*
- Health system coordination, public communication encouraging increased hygiene and personal protection, case isolation, mild social distancing measures (large public gatherings banned, work from home where possible, social distancing recommended) - *Sweden's policy*
- Health system coordination, public communication encouraging increased hygiene and personal protection, case isolation, mild social distancing measures (large public gatherings banned, work from home where possible, social distancing recommended), contact tracing involving publicly disclosed detailed location information of individuals that tested positive for COVID-19 - *South Korea's policy*
- Health system coordination, public communication encouraging increased hygiene and personal protection, case isolation, mild social distancing measures (large public gatherings banned, work from home where possible, social distancing recommended), complete physical isolation of the elderly from other age groups for 12 weeks - *Enhanced distancing for vulnerable groups*
- Health system coordination, public communication encouraging increased hygiene and personal protection, case isolation, strict social distancing measures (large public gatherings banned, work from home where possible, school and university closures, restaurants and shopping malls closures, restricted mobility) - *Lockdown*

6. Is there any other set of measures you believe would have been better than any of the ones described at Question 5? Briefly indicate what that would be.

7. Which are, in your view, the main concerns to be addressed by governmental responses to the SARS-CoV-2 pandemic? Drag the slider scale using the mouse to specify what has more priority for you:

i. Health and epidemiologic aspects

ii. Economic aspects

iii. Social and behavioral aspects

iv. Environmental aspects

v. Political and governance aspects

8. What health problems do you think are more important to limit during a SARS-CoV-2 epidemic in Romania? Drag the slider scale using the mouse to specify what should be a priority to address by mitigation actions:

i. Reducing the number of direct COVID-19 fatalities

ii. Reducing the number of indirect fatalities, caused by healthcare, economic or social problems brought by the epidemic

9. Which are, in your view, the most important economic aspects to be addressed in a pandemic response action? Drag the slider scale using the mouse to specify which issues have more priority for you:

i. The short-term costs of the pandemic

ii. Unemployment

iii. Taxes and budget

iv. Specific industries affected

v. Growing industries

10. Which are, in your view, the most important social aspects to be addressed in a pandemic response action? Drag the slider scale using the mouse to specify which issues have more priority for you:

i. Preserving human rights

ii. Protecting vulnerable groups

iii. Avoiding the increase of criminality rates

iv. Preserving mental health

v. Ensuring access to education and training

11. Which are, in your view, the most important political aspects to be addressed in a pandemic response action? Drag the slider scale using the mouse to specify which issues have more priority for you:

i. Reducing the risk of short-term governmental abuse

ii. Ensuring citizen approval of measures to be taken

iii. Maintaining or increasing trust in government

iv. Increasing resilience and preparedness for hazard events

12. What is your opinion on the following statements? Choose the option which is closest to your view.

☐completely disagree	☐somewhat disagree	☐ I neither agree, nor disagree	☐ somewhat agree	☐completely agree

- The number of direct COVID-19 fatalities is more important than the effect of climate change and pollution on people’s health in Romania.
- The number of direct COVID-19 fatalities is more important than unemployment levels in Romania.
- The number of direct COVID-19 fatalities is more important than mental health.
- The number of direct COVID-19 fatalities is more important than the risk of short-term governmental abuses.
- The number of direct COVID-19 fatalities is more important than ensuring access to education for children and young students.
- The number of indirect fatalities is more important than the citizen approval of governmental action in response to the pandemic.
- The short term costs of the measures taken by the government are more important than the citizen approval of governmental action in response to the pandemic.
- The protection of vulnerable groups is more important than the risk of short-term governmental abuses.
- The protection of vulnerable groups is more important than the economic impact on specific industries.
- The number of direct COVID-19 fatalities is more important than the preservation of human rights.

13. Which are the main sources you use for information on COVID-19 related issues? Select the frequency of use from 1 - most often, 2 - often, 3 - sometimes, 4 - rarely, 5 - never.

TV	☐1	☐2	☐3	☐4	☐5
Radio	☐1	☐2	☐3	☐4	☐5
Print newspapers	☐1	☐2	☐3	☐4	☐5
National online news platforms	☐1	☐2	☐3	☐4	☐5
International online news platforms	☐1	☐2	☐3	☐4	☐5

Social media	☐1	☐2	☐3	☐4	☐5
National institutional websites	☐1	☐2	☐3	☐4	☐5
International institutions' websites	☐1	☐2	☐3	☐4	☐5
Academic outlets	☐1	☐2	☐3	☐4	☐5
NGOs	☐1	☐2	☐3	☐4	☐5

14. How much do you trust the following sources of information? Select for each whether you have 1 - complete trust, 2 - some trust, 3 - little trust, 4 - very little trust, 5 - no trust.

TV	☐1	☐2	☐3	☐4	☐5
Radio	☐1	☐2	☐3	☐4	☐5
Print newspapers	☐1	☐2	☐3	☐4	☐5
National online news platforms	☐1	☐2	☐3	☐4	☐5
International online news platforms	☐1	☐2	☐3	☐4	☐5
Social media	☐1	☐2	☐3	☐4	☐5

National institutional websites	☐1	☐2	☐3	☐4	☐5
International institutions' websites	☐1	☐2	☐3	☐4	☐5
Academic outlets	☐1	☐2	☐3	☐4	☐5
NGOs	☐1	☐2	☐3	☐4	☐5
