# Supplementary material for: A Multi-Criteria Framework for Pandemic Response Measures
Source: Front Public Health. 2021 Apr 20;9:583706. doi: 10.3389/fpubh.2021.583706 (PMC8096778; doi:10.3389/fpubh.2021.583706)
Supplement: Supplementary file 2 [file Data_Sheet_2.pdf]

Appendix 2 - AnyLogic input parameters:

Epidemiologic parameters:

- Infected (days): Number of days an individual is infected and infectious. 5.0
- Exposed (days): Number of days between an individual gets infected and becomes infectious. 5.1

Infectivity:

- Infectivity (% of infectiousness): The reduction in % of infectiousness for asymptomatic, mild, severe, and critical cases. 50.0, 0.0, 0.0, 0.0

Total reported cases 3 Jan 2021: 643,559

Total deaths: 16,057
95.2% of deaths were of patients with comorbidities
84.7% of deaths were in the 60+ age group

- Total cases / age group:

0-19 years: 7%
20-59 years: 66%
60+ years: 27%

- Total deaths / age group:

0-19 years: 0%
20-59 years: 14%
60+ years: 86%

- Population size: 19,370,448

0-19 years: 4,063,856 - 20.98%
20-59 years: 10,354,630 - 53.46%
60+ years: 4,951,962 - 25.56%

- Incidence rate:

14-day case notification rate per 100 000 inhabitants: 253.08
14-day death notification rate per 100 000 inhabitants: 8.16

Data sources for epidemiologic evolution and demography in Romania: European Center for Disease Control, National Institute of Public Health Romania and National Institute of Statistics
